# Applying productivity and phytonutrient profile criteria in modelling species selection of microgreens as Space crops for astronaut consumption

**DOI:** 10.3389/fpls.2023.1210566

**Published:** 2023-08-11

**Authors:** Luigi Gennaro Izzo, Christophe El Nakhel, Youssef Rouphael, Simona Proietti, Gabriele Paglialunga, Stefano Moscatello, Alberto Battistelli, Maurizio Iovane, Leone Ermes Romano, Stefania De Pascale, Giovanna Aronne

**Affiliations:** ^1^ Department of Agricultural Sciences, University of Naples Federico II, Portici, Italy; ^2^ National Research Council of Italy, Research Institute on Terrestrial Ecosystems, Porano, Italy

**Keywords:** functional food, phytonutrients, space crops, space food, species selection, *Raphanus sativus*, *Brassica oleracea* var. *capitata* f. *sabauda*

## Abstract

**Introduction:**

Long-duration missions in outer Space will require technologies to regenerate environmental resources such as air and water and to produce food while recycling consumables and waste. Plants are considered the most promising biological regenerators to accomplish these functions, due to their complementary relationship with humans. Plant cultivation for Space starts with small plant growth units to produce fresh food to supplement stowed food for astronauts’ onboard spacecrafts and orbital platforms. The choice of crops must be based on limiting factors such as time, energy, and volume. Consequently, small, fast-growing crops are needed to grow in microgravity and to provide astronauts with fresh food rich in functional compounds. Microgreens are functional food crops recently valued for their color and flavor enhancing properties, their rich phytonutrient content and short production cycle. Candidate species of microgreens to be harvested and eaten fresh by crew members, belong to the families Brassicaceae, Asteraceae, Chenopodiaceae, Lamiaceae, Apiaceae, Amarillydaceae, Amaranthaceae, and Cucurbitaceae.

**Methods:**

In this study we developed and applied an algorithm to objectively compare numerous genotypes of microgreens intending to select those with the best productivity and phytonutrient profile for cultivation in Space. The selection process consisted of two subsequent phases. The first selection was based on literature data including 39 genotypes and 25 parameters related to growth, phytonutrients (e.g., tocopherol, phylloquinone, ascorbic acid, polyphenols, lutein, carotenoids, violaxanthin), and mineral elements. Parameters were implemented in a mathematical model with prioritization criteria to generate a ranking list of microgreens. The second phase was based on germination and cultivation tests specifically designed for this study and performed on the six top species resulting from the first ranking list. For the second selection, experimental data on phytonutrients were expressed as metabolite production per day per square meter.

**Results and discussion:**

In the final ranking list radish and savoy cabbage resulted with the highest scores based on their productivity and phytonutrient profile. Overall, the algorithm with prioritization criteria allowed us to objectively compare candidate species and obtain a ranking list based on the combination of numerous parameters measured in the different species. This method can be also adapted to new species, parameters, or re-prioritizing the parameters for specific selection purposes.

## 1 Introduction

Moving toward long-duration missions in Space, it will be vital to grow healthy plant food intended for astronaut consumption and to regenerate resources during spaceflights and extraterrestrial colonization (Dueck et al., 2016; De Pascale et al., 2021). Even more, considering that pre-packaged food has been shown to not meet the shelf-life requirements of long-duration scenarios (Cooper et al., 2017; Carillo et al., 2020), it is mandatory to develop Bio-regenerative Life-Support Systems (BLSS) that will ultimately replace also the need for food resupply from Earth (Dueck et al., 2016). MELiSSA (Micro-Ecological Life Support System Alternative), the European Space Agency program, seeks to develop BLSSs with plant compartments to support human life for long crewed Space missions (Carillo et al., 2020). BLSSs implementation starts with small plant growth units (i.e., salad machines) to produce some fresh foods to supplement stowed food for astronauts onboard the International Space Station or during early lunar missions. The cultivation area will then be expanded for longer duration lunar missions, which will also provide an opportunity to test systems and procedures for Mars missions, where BLSSs would play a more crucial role (Wheeler, 2017). In the last decades, various plant cultivation systems in Space have been developed such as Veggie and the Advanced Plant Habitat (APH) (Zabel et al., 2016). Specifically, Veggie is a small plant growth chamber designed by NASA to produce fresh vegetables onboard the International Space Station (ISS) and is currently providing fresh food as supplement for astronaut diet (Massa et al., 2016). APH is also currently working on the ISS with a higher technological level compared to Veggie, for purposes more related to research studies on plant growth in Space conditions. In this framework, the Italian Space Agency (ASI), in collaboration with the University of Naples Federico II, the University of Tor Vegata, the Italian National Research Council (CNR) and the “Agenzia nazionale per le nuove tecnologie’’ (ENEA), is currently developing novel systems and technologies intended for microgreens production in Space.

Although much effort has been devoted to the design and development of cultivation systems in Space, only a few studies provide a selection of the best species/cultivars to be used in flight experiments or cultivations in Space. For example, De Micco et al. (2012) developed an objective and repeatable methodology for the selection of soybean cultivars to be used in BLSSs. A specific algorithm considering the relevance of the different plant traits was elaborated to rank the soybean cultivars and to identify the best ones for subsequent cultivation trials. Similarly, Massa et al. (2015) reported a selection of leafy greens vegetables as supplement astronaut diet to be grown in the Veggie growth chamber onboard the ISS by using a ranking method based on growth, nutrition, and organoleptic parameters. Dueck et al. (2016) also reported a selection methodology for choosing Space crops using literature data and information provided by growers on horticultural aspects, human nutrition, and psychology, and precluding the selection of crops that are not ready to eat. A further study by Aronne et al. (2020) was aimed to select the best species among 50 candidate crops to conduct an experiment onboard the ISS using refurbished hardware. Authors applied subsequent inclusion/exclusion criteria to select the biological system that best fit to the hardware and timing expected during the prelaunch, launch and flight operations (Aronne et al., 2020).

Overall, the selection of plant species for cultivation in Space is effort demanding since there are specific constraints and Space factors to be addressed in the different mission scenarios. Microgreens are functional food crops recently valued for their flavor and phytonutrient content and have been proposed to produce fresh food to supplement stowed food for astronauts during spaceflight or onboard orbital platforms since they can be harvested and eaten fresh by crew members (Kyriacou et al., 2016; Kyriacou et al., 2017). As candidate crops for plant production system in Space, microgreens represent a source of vitamins, minerals and antioxidant which are often lacking in non-perishable or frozen food but fundamental to maintain a healthy status of the crew members and counteract negative effects due to the spaceflight environment (Gómez et al., 2021). Microgreens may be considered a resilient phytochemical factory with the potential to provide a significant nutritional addition in just a small serving size so they can be used to meet dietary needs of the astronauts by reaching targeted daily intakes of specific nutraceuticals and reducing the need for large storage volumes and the related costs. Overall, the plant species to be used for microgreens production is the first factor influencing the availability and the content level of specific phytonutrients (Teng et al., 2022). Candidate genotypes of microgreens mostly include species from the Brassicaceae, Asteraceae, Chenopodiaceae, Lamiaceae, Apiaceae, Amarillydaceae, Amaranthaceae, and Cucurbitaceae families (Caracciolo et al., 2020; Ebert, 2022).

As explained by Kyriacou et al. (2021), microgreens other than being a gastronomic novelty, they gather appreciable characteristics such as compact shape, fast production, and confined space requirement. Moreover, microgreens easily fit the criteria cited for Space farming such as plant size, harvest index, light requirement, nutritional value (Dueck et al., 2016; Carillo et al., 2020), volume/energy efficiency, disease resistance, and handling time (Dueck et al., 2016). As reported by Carillo et al. (2020), the choice of the crop should consider its amount of biologically active compounds that contribute to psychological wellbeing and health promotion of astronauts. However, to the authors’ knowledge no previous studies have reported a selection of microgreens to be used as functional Space food, and there is a need to identify those species/cultivars with satisfactory yield and food quality. In this context, we developed a selection method for microgreens species, based on an objective comparison of numerous genotypes aiming to identify those with the best growth and nutritional characteristics for cultivation in Space. Herein we describe the algorithm used to generate the ranking list by adopting a two-level selection based on growth and nutritional parameters, and their prioritization.

## 2 Materials and methods

### 2.1 Literature survey and data elaboration

For the first round of selection, we conducted a literature survey and evaluated several articles reporting data on microgreens. To obtain a reliable dataset for comparing the different species, we decided to use data only from those articles reporting a comparison between several microgreens’ species grown under the same conditions. Specifically, we used data provided by Xiao et al. (2012); Xiao et al. (2016), and Kyriacou et al. (2019). In this regard, the studies by Xiao et al. (2012) and Xiao et al. (2016) were conducted in unheated greenhouses and under ambient light, whereas Kyriacou et al. (2019) conducted their trials under controlled environment using commercial peat-based substrate fertigated with a quarter-strength Hoagland and Arnon formulation, day/night temperatures of 22/18 ± 2°C, 12 h photoperiod, relative air humidity of 65-75% and photosynthetic photon flux density (PPFD) of 300 ± 10 μmol m^-2^ s^-1^. With this approach we gathered data on 39 genotypes (**Table 1**) and 25 parameters included in 3 categories (**Table 2**), that were used to construct a working matrix for the first algorithm elaboration. To compare the 39 genotypes of microgreens, data of each parameter were normalized considering as 0 and 1 the minimum and maximum values, respectively, measured for that parameter. Specifically, as also reported in Massa et al. (2015), average values of each parameter were normalized among cultivars/species so that the minimum value equates to 0 and the maximum value equates to 1 using the following formula: X_i_ = [x_i_ – min(x)/(max(x) – min(x)]. As regards those parameters for which lower values are most desirable (i.e., sowing density, growth period, seed weight, nitrate, and iron), the normalized data were inverted so that the minimum value was equal to 1 and the maximum value was equal to 0.

**Table 1:** List of microgreens genotypes used for the selection.

N	Family	Genus	Species	Variety	Common name
1	Amaranthaceae	*Amaranthus*	*hypochondriacus*		Amaranth
2	Apiaceae	*Apium*	*graveolens*		Celery
3	Apiaceae	*Coriandrum*	*sativum*		Coriander
4	Brassicaceae	*Barbarea*	*verna*		Cress
5	Brassicaceae	*Brassica*	*juncea*		Brown mustard
6	Brassicaceae	*Brassica*	*napus*	*napobrassica*	Rutabaga
7	Brassicaceae	*Brassica*	*oleracea*	*acephala*	Black cabbage
8	Brassicaceae	*Brassica*	*oleracea*	*alboglabra*	Chinese kale
9	Brassicaceae	*Brassica*	*oleracea*	*botrytis*	Cauliflower
10	Brassicaceae	*Brassica*	*oleracea*	*capitata* f. *alba*	White cabbage
11	Brassicaceae	*Brassica*	*oleracea*	*capitata* f. *rubra*	Red cabbage
12	Brassicaceae	*Brassica*	*oleracea*	*capitata* f. *sabauda*	Savoy cabbage
13	Brassicaceae	*Brassica*	*oleracea*	*gongylodes*	Kohlrabi
14	Brassicaceae	*Brassica*	*oleracea*	*italica*	Broccoli
15	Brassicaceae	*Brassica*	*oleracea*	*pekinensis*	Napa cabbage
16	Brassicaceae	*Brassica*	*rapa*	*chinensis*	Pak choy
17	Brassicaceae	*Brassica*	*rapa*	*gemmifera*	Brussels sprouts
18	Brassicaceae	*Brassica*	*rapa*	*narinosa*	Tatsoi
19	Brassicaceae	*Brassica*	*rapa*	*nipposinica*	Mizuna
20	Brassicaceae	*Brassica*	*rapa*	*perviridis*	Komatsuna
21	Brassicaceae	*Brassica*	*rapa*	*rapa*	Turnip
22	Brassicaceae	*Brassica*	*rapa*	*ruvo*	Rapini
23	Brassicaceae	*Eruca*	*sativa*		Rocket
24	Brassicaceae	*Lepidium*	*bonariense*		Peppercress
25	Brassicaceae	*Lepidium*	*sativum*		English cress
26	Brassicaceae	*Nasturtium*	*officinale*		Watercress
27	Brassicaceae	*Raphanus*	*sativus*	*longipinnatus*	Daikon radish
28	Brassicaceae	*Raphanus*	*sativus*		Radish
29	Brassicaceae	*Wasabia*	*japonica*		Wasabi
30	Chenopodiaceae	*Atriplex*	*hortensis*		Garden orache
31	Chenopodiaceae	*Beta*	*vulgaris*		Beet
32	Chenopodiaceae	*Spinacia*	*oleracea*		Spinach
33	Fabaceae	*Pisum*	*sativum*		Pea
34	Lamiaceae	*Ocimum*	*basilicum*	*purpurascens*	Red rubin basil
35	Lamiaceae	*Ocimum*	*basilicum*		Basil
36	Malvaceae	*Corchorus*	*olitorius*		Jute mallow
37	Poaceae	*Zea*	*mays*		Maize
38	Polygonaceae	*Rumex*	*acetosa*		Sorrel
39	Polygonaceae	*Rumex*	*acetosella*		Red sorrel

**Table 2:** Priority levels of categories and parameters used for the first ranking of microgreens.

Category	Category priority (P)	Parameter	Parameter priority (p)
Growth	2	Yield	5
		Dry weight	4
		Growth period	3
		Sowing density	1
		Seed weight	1
Phytochemicals	3	Ascorbic acid (Vitamin C)	5
		Polyphenols	5
		Tocopherol (Vitamin E)	5
		β-carotene	5
		Antioxidant activity	2
		Phyllochinon (Vitamin K)	2
		Lutein	2
		Violaxantin	2
		Chlorophylls	2
Elements	1	Calcium	5
		Phosphorus	3
		Magnesium	3
		Nitrate	3
		Potassium	3
		Iron	3
		Sodium	2
		Sulfur	2
		Manganese	2
		Copper	2
		Zinc	2

### 2.2 Assignment of priority levels to categories and parameters in the first selection phase

For the selection method we considered the relative importance of the different categories and parameters in the framework of cultivation in Space. Specifically, we assigned values ranging between 1-3 for categories and 1-5 for parameters (**Table 2**). As regards categories, we set the highest priority (P = 3) to “Phytochemicals”, and then descending to “Growth” (P = 2) and to “Elements” (P = 1). As regards parameters included in the “Growth” category we assigned the highest values to yield (p = 5) and dry weight (p = 4) and then in descending order growth period (p = 3), sowing density (p = 1) and seed weight (p = 1) (**Table 2**). As regards the parameters related to quality aspects included in the categories “Phytochemicals” and “Elements” we assigned the specific priority levels considering nutritional requirements, physiological level content, and diet composition defined for Space missions (Smith et al., 2014; Smith et al., 2015). To quantify the priority values to be attributed to different parameters, three criteria were mainly considered. First, the nutraceutical importance of the compound and its effects due to the intake on human health such as the role in the metabolism, in the absorption of nutrients, the antioxidant and anti-inflammatories power and that of contrasting specific illness of the astronauts. As a second criterion we considered the loss of the putative component of the astronaut physiological level during the flight to select those species that can supply adequate amounts of phytonutrients to counterbalance the loss. Third, the presence of the different compounds in the astronaut diet and the perishability of Space food due to the Spacecraft environment. The low availability or absence of the specific nutraceutical compound in Space food, as well its degradation due to flight environmental factors such as temperature, atmosphere and radiation resulted in an important characteristic to be considered, especially in concomitance of a high nutraceutical role of the component). With this approach we assigned the highest priority level (p = 5) to ascorbic acid, β-carotene, tocopherol, and total polyphenols since their relevance for daily consumption was recognized. Lower priority (p = 2) was assigned to the remaining variables of the “Phytochemicals” category. As “Elements” category, the highest priority was assigned to calcium (p = 5), whereas an intermediate value (p = 3) was assigned to phosphorus, potassium, and magnesium. Finally, we assigned a value of p = 2 to sodium, sulfur, manganese, copper, and zinc. For both nitrate and iron (inverted value), which were considered as not desirable parameters due to their negative effects on human health, we assigned a value of p = 3 (**Table 2**).

### 2.3 Algorithm calculation

For each microgreens genotype, the formula (1) was used to calculate the score of the individual parameters (*s*_i_) as the product of the normalized value of the parameter (x_i_) and its priority level (*p*_i_):

(1)


$$s_{i}=\;x_{i}\times p_{i}$$

The formula (2) was then used to calculate the score of the individual categories (X_i_) as the average of the scores of the parameters included in the category:

(2)


$$X_{i}=\;µ\;\left(s_{i}\right)$$

Finally, the formula (3) was used to calculate the score of each genotype of microgreens (*S*_i_) as the sum of the products between the scores of the categories (X_i_) and their respective priority levels (*P*_i_):

(3)


$$S_{i}=\;\Sigma \;\left(X_{i}\times P_{i}\right)$$

Based on the calculated scores, we obtained a first ranking list of microgreens (Results, **Table 3**).

**Table 3:** Ranking list of microgreens based on the scores calculated using literature data.

Rank	Species	Common name	Score
1	*Coriandrum sativum*	Coriander	4.687
2	*Brassica oleracea* var. *capitata* f. *sabauda*	Savoy cabbage	4.632
3	*Raphanus sativus* var. *longipinnatus*	Daikon radish	4.586
4	*Brassica oleracea* var. *capitata* f. *rubra*	Red cabbage	4.541
5	*Brassica oleracea* var. *capitata* f. *alba*	White cabbage	4.487
6	*Raphanus sativus*	Radish	4.375
7	*Brassica oleracea* var. *italica*	Broccoli cabbage	4.373
8	*Brassica oleracea* var. *acephala*	Black cabbage	4.232
9	*Brassica oleracea* var. *alboglabra*	Chinese kale	4.214
10	*Brassica oleracea* var. *botrytis*	Cauliflower	4.209
11	*Brassica rapa* var. *ruvo*	Rapini	4.203
12	*Brassica oleracea* var. *pekinensis*	Napa cabbage	4.053
13	*Brassica oleracea* var. *gongylodes*	Kohlrabi	4.035
14	*Brassica rapa* var. *narinosa*	Tatsoi	3.829
15	*Brassica rapa* var. *chinensis*	Pak choy	3.746
16	*Brassica rapa* var. *perviridis*	Komatsuna	3.720
17	*Brassica rapa* var. *rapa*	Turnip	3.693
18	*Brassica napus* var. *napobrassica*	Rutabaga	3.673
19	*Brassica rapa* var. *nipposinica*	Mizuna	3.523
20	*Brassica rapa* var. *gemmifera*	Brussels sprouts	3.434
21	*Ocimum basilicum*	Basil	3.181
22	*Ocimum basilicum* var. *purpurascens*	Red rubin basil	3.162
23	*Beta vulgaris*	Beet	3.024
24	*Amaranthus hypochondriacus*	Amaranth	2.974
25	*Brassica juncea*	Brown mustard	2.872
26	*Wasabia japonica*	Wasabi	2.714
27	*Lepidium sativum*	English cress	2.701
28	*Lepidium bonariense*	Peppercress	2.658
29	*Eruca sativa*	Rocket	2.500
30	*Rumex acetosella*	Red sorrel	2.376
31	*Corchorus olitorius*	Jute mallow	2.250
32	*Pisum sativum*	Pea	2.022
33	*Apium graveolens*	Celery	1.550
34	*Barbarea verna*	Cress	1.514
35	*Atriplex hortensis*	Garden orache	1.272
36	*Nasturtium officinale*	Watercress	1.209
37	*Spinacia oleracea*	Spinach	1.050
38	*Zea mays*	Maize	0.972
39	*Rumex acetosa*	Sorrel	0.956

### 2.4 Second selection phase

The elaboration carried out for the first level of selection provided the ranking list based on the scores of 39 microgreens genotypes. From this ranking list, we selected the 6 top species for the second level of selection, namely coriander, savoy cabbage, daikon radish, red cabbage, white cabbage, and common radish. Differently from the first phase, in the second phase of selection we used an experimental approach based on germination and cultivation tests to gather similar and additional data to those collected from the literature for the first selection.

#### 2.4.1 Germination tests and seed volume

The six top species (i.e., coriander, savoy cabbage, daikon radish, red cabbage, white cabbage, and common radish) were analyzed to evaluate germination rates at different days after sowing (DAS). Seeds of *Brassica oleracea* var. *capitata* f. *sabauda* cv. Vertus, *Brassica oleracea* var. *capitata* f. *alba* cv. Copenhagen 2, *Brassica oleracea* var. *capitata* f. *rubra* cv. Cabeza Negra 2, *Raphanus sativus* var. *longipinnatus* cv. White Icicle, and *Raphanus sativus* cv. Saxa 2 were provided by Pagano Costantino & F.lli Srl (Scafati, Salerno, Italy). Seeds of *Coriandrum sativum* L. cv. Micro Splits were provided by CN Seeds Ltd., Pymoor, Ely, Cambrigeshire, UK. For each species, the germination tests were conducted in an incubator at 22 ± 0.5°C using four Petri dishes lined with laboratory filter paper wetted with distilled water. 100 seeds were placed in each Petri dish, for a total of 400 seeds per species. The percentage of germination was evaluated at 3, 5, and 10 days after sowing.

For each of the species tested, seed volume was measured using a 10 mL graduated cylinder containing distilled water. In particular, 100 seeds were submersed in 3 mL of water inside the cylinder and the volume of the seeds was measured as an increase in the total volume.

#### 2.4.2 Cultivation tests - plant material and growth conditions

Cultivation tests were conducted using the same seeds of coriander, savoy cabbage, daikon radish, red cabbage, white cabbage, and common radish used for the germination tests. Based on the size of the different seeds, a sowing density of 4 seeds cm^-2^ was adopted for coriander and the genus *Raphanus*, and 5 seeds cm^-2^ for the genus *Brassica*. The microgreens seeds were sown in plastic trays (∼200 cm^2^) filled with peat-based substrate (Special Mixture, Floragard Vertriebs-GmbH, Oldenburg, Germany; pH 5.48 and EC 0.282 mS cm^-1^) and placed in a fully controlled growth chamber (KBP-6395F, Termaks, Bergen, Norway). Seed germination occurred in darkness at 24°C and 100% relative humidity (RH) for six days for coriander and four days for the rest. During germination, the seeds were sprayed with osmotic water (EC = 100 ± 10 µS cm^-1^), whereas after emergence, plants were fertigated with a quarter-strength Hoagland solution (pH = 5.9 ± 0.2 and EC = 400 ± 25 µS cm^-1^). Once the microgreens emerged, the artificial light (400-700 nm) provided by light-emitting diode panels (K5 Series XL750, Kind LED, Santa Rosa, CA, USA) was turned on (12h photoperiod), and the growth chamber was set at 24/18 ± 2°C and RH 65 ± 5%. The LED panels were set to deliver an adequate absorption spectrum for photosynthesis, and an average photosynthetic photon flux density (PPFD) of 300 ± 15 µmol m^-2^ s^-1^ at canopy level. Each cultivar cultivation was replicated three times (tray = replicate), and all the trays were rotated daily within the growth chamber to maintain a homogeneous light, temperature, and humidity repartition.

#### 2.4.3 Cultivation tests - colorimetric measurement, harvest and sampling

**Figure 1:**
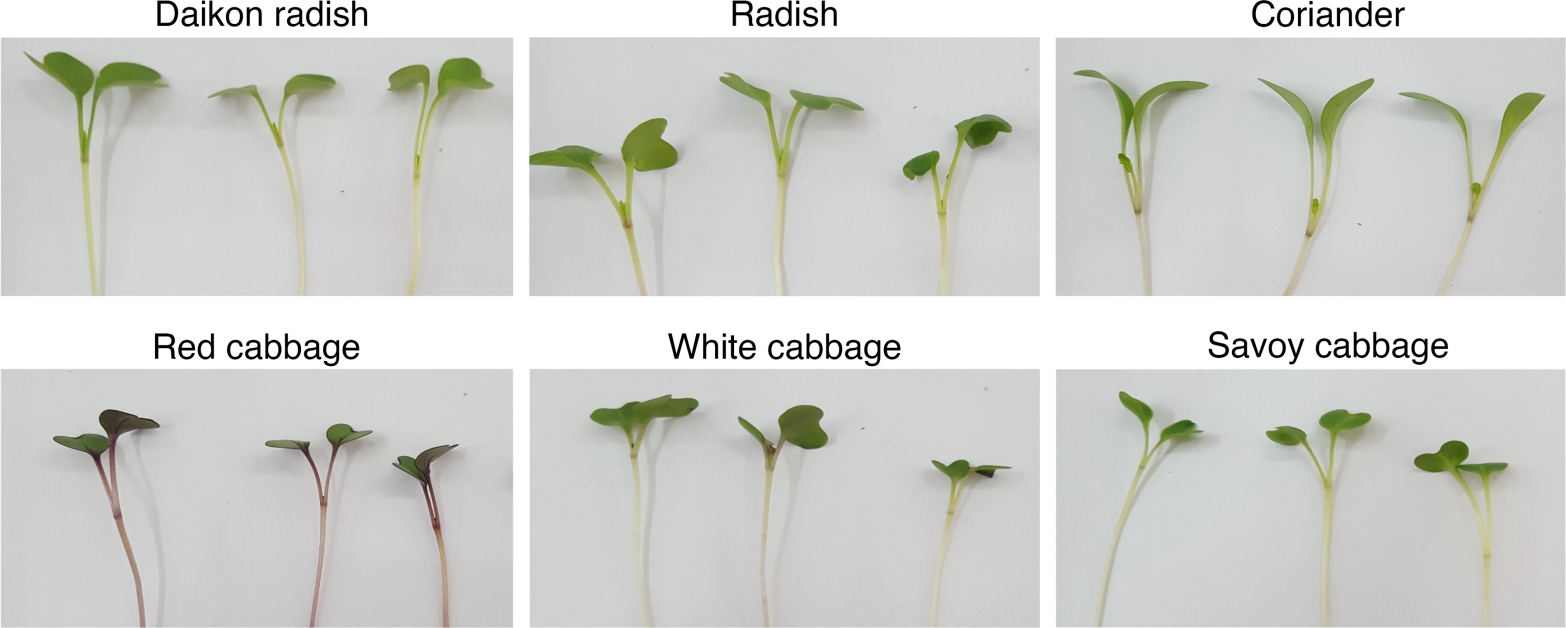
Representative photos of the cotyledonary stage of the different microgreens’ species at harvest.

Just before harvest, the CIELAB color space parameters (L*, a* and b*) of microgreens canopy were measured by taking eight measurements per tray *via* a portable Minolta Chroma meter (CR-400, Minolta Co. Ltd., Osaka, Japan). Then a* and b* values were used to calculate the hue angle and chroma. For harvest, cotyledonary stage was chosen for all the cultivars, as shown in **Figure 1**, resulting in 13, 14 and 16 days after sowing (DAS) for *Raphanus* genus, *Brassica* genus and coriander, respectively. All microgreens’ cultivars were harvested just above the substrate level by avoiding any impurities, and they were assessed directly for their fresh yield that was expressed in kg m^-2^. Eight randomly chosen microgreens plantlets per tray were measured to assess hypocotyl length, which was expressed in cm. Part of the harvested material was placed in a forced air oven at 60°C until constant weight, in order to assess the dry weight of each replicate and consecutively calculate dry matter (DM) %. The remaining harvested material was directly placed in liquid nitrogen, and then stored at -80°C to be used successively for the qualitative analysis.

#### 2.4.4 Cultivation tests - non-structural carbohydrates

Measurements of non-structural carbohydrates (NSC) were performed using 10 mg samples of the powder obtained from the freeze-dried material. The extraction was performed in 1 mL of 80% ethanol at 80°C for 45 min under continuous shaking conditions. The extract was centrifuged at 16000*g* for 5 min, soluble sugars (glucose, fructose, and sucrose) were recovered in the supernatant, and starch was in the pellet. Soluble sugar determination, by spectrophotometric coupled enzymatic assay, was performed, as described in Scartazza et al. (2017). All sugar assays were performed in dual-wavelength mode (340–405 nm) in a plate reader (Spectrotar Nano BMG, Labatech Gmbh, Ortenberg, Germany). The pellet, containing starch, was washed four times with a 50 mM NaAcetate buffer (pH 4.5) and then suspended and autoclaved at 120°C for 45 min in 1 mL of the same buffer. After autoclaving, the sample was incubated at 50°C for 1 h with amyloglucosidase (70 U) and α-amylase (4U) to hydrolyze the starch to glucose. The glucose produced by starch hydrolysis was then measured as described before by spectrophotometric coupled enzymatic assay.

#### 2.4.5 Cultivation tests - pigments content

Neoxanthin, violaxanthin, β-carotene, and lutein content were determined by the extraction of 10 mg powdered freeze-dried microgreens with 2 mL 100% acetone at 4°C under dark conditions using a glass-glass homogenizer. The samples were centrifuged at 16000*g* for 5 min at 4°C and filtered through a 0.2 μm nylon PPII syringe disposable filter; 15 μL of the clear extract was used to determine the concentration of pigments by an HPLC U3000 system (Dionex™ ICS-5000; Thermo Fisher Scientific, Waltham, MA, United States), equipped with a C18(2) LUNA (Phenomenex, Bologna, Italy) analytical column (5 μm, 250 mm × 4.6 mm) and a related guard column (Phenomenex, Bologna, Italy) maintained at 30°C. All separations were achieved isocratically using, from 0 to 4 min, a mobile phase composed of solution A: 1.75% water, 1.75% methanol, 1.75% dichloromethane, and 94.75% acetonitrile, and from 4.1 to 18 min a mobile phase composed of solution B: 50% acetonitrile and 50% diethyl acetate, with a final re-equilibration of 4 min with solution A. The flow rate was 1 ml min^−1^ for a total run time of 22 min. The autosampler was maintained at 4°C, the UV detector wavelength was set at 440 nm, and concentrations of pigments were determined against standard curves (Žnidarčič et al., 2011). Chlorophylls (Chl a and Chl b) were quantified spectrophotometrically using the same ethanolic extracts used for NSC determination, as described in Lichtenthaler and Wellburn (1983).

#### 2.4.6 Cultivation tests - total anthocyanins and total phenolic content

Total anthocyanins were determined by extracting 10 mg of lyophilized powder in 2 ml of 1% HCl in methanol for 1 hour at 65°C. The liquid extract was separated by centrifugation at 16000*g* for 5 minutes. After centrifugation, the supernatant was separated, and the total anthocyanin content was quantified spectrophotometrically by measuring the absorbance at 530 nm and 657 nm to correct the chlorophylls degradation products. Concentration was expressed as cyanidin-3-glucoside equivalent values and using an extinction coefficient of 30000 mol^-1^ cm^-1^. The phenolic component (Total Polyphenol Content, TPC) was quantified according to the protocol described by Usenik et al. (2008) by extracting 10 mg of powder from the lyophilized samples in 2 ml of 100% methanol. After centrifugation at 16000*g* for 5 min, the supernatant was recovered and used for the spectrophotometric quantification of polyphenols, determining the absorbance at 765 nm. The amount of total polyphenols was then calculated by relating the absorbance of each sample to the calibration curve of gallic acid.

#### 2.4.7 Cultivation tests – total ascorbic acid

For the determination of total ascorbic acid (Tot. Asc. A.), 10 mg of the frozen powder was extracted in an ice-cold glass-glass homogenizer with 2 mL of 3% Metaphosphoric acid (MPA) at 4°C. The mixtures were centrifuged at 16000 × g for 5 min at 4°C. The supernatants were filtered through a 0.2 µm (Whatman) PPII nylon filters. Tris (2-carboxyethil) phosphine (TCEP)was added as a reducing agent to the final concentration of 5 mmol/L in the filtered extract, which was then incubated for 30 min at 25°C in order to reduce all the dehydro-ascorbic acid to ascorbic acid. After 30 minutes, 5 µl of samples were injected into HPLC for the quantification of Tot. Asc. A. The chromatographic method used is that described in Chebrolu et al. (2012) with minimal changes. Tot. Asc. A. was analyzed using an UltiMate 3000 HPLC System ThermoScientific™ Dionex (Sunnyvale, CA, USA) coupled with a UV/VIS detector (ThermoScientific™ Dionex). The separation was performed using a Phenomenex Luna C18(2) column (250 mm × 4.6 mm i.d. and particle size 5 μm) and the run time was 15 min. The Tot. Asc A. peak was detected at 254 nm and the processing of the chromatographic peaks was performed using the software version Chromeleon 7.2 (ThermoScientific ™ Dionex). The entire chromatographic separation was performed in an isocratic mobile phase consisting of 0.010 mol L^-1^ of KH_2_PO_4_, maintained at pH 2.8 and flow rate of 0.7 mL min^-1^. The quantification was performed by means of a calibration curve of an ascorbic acid standard. All the reagents used are of a high degree of purity for HPLC analysis.

#### 2.4.8 Cultivation tests - nitrate, phosphate, and sulfate

The determination of inorganic anions was performed by the extraction of 10 mg of powdered materials in water at 80°C for 45 min under continuous shaking conditions. The extract was centrifuged at 16000*g* for 5 min and the supernatants were filtered through a 0.2 μm nylon PPII syringe filter prior to injection on an ion chromatography system (Dionex™ ICS-5000; Thermo Fisher Scientific, Waltham, MA, United States) equipped with a conductivity detector, an analytical IonPac AS11-HC column (4 × 250mm) (Thermo Fisher Scientific, Waltham, MA, United States) with a related guard column and an IonPac Anion Trap Column (ATC)-1 (Thermo Fisher Scientific, Waltham, MA, United States). The system was coupled with an ERSTM 500 Electrolytically Regenerated Suppressor (Dionex™ ICS-5000; Thermo Fisher Scientific, Waltham, MA, United States) applying the external water mode configuration, using 100% of methanol. Runs were carried out at 30°C and a flow rate of 1ml min^−1^ using a sodium hydroxide stepped gradient as reported by Proietti et al. (2019). The electrical signal was integrated into micro-Siemens (μS). The eluents and the inorganic anion standard solutions were prepared using HPLC-grade reagents (Merck KGaA, Darmstadt, Germany). U3000-HPLC and ICS-5000 chromatography system control, data acquisition, and processing were performed with the software Chromeleon Data System 6.8 (Dionex™ ICS-5000; Thermo Fisher Scientific, Waltham, MA, United States).

#### 2.4.9 Assignment of priority levels to categories and parameters in the second selection phase

Data obtained from the germination, cultivation, and quality tests were entered in the working matrix and used to calculate the ranking list of the 6 top species using a similar procedure as described for the first algorithm elaboration. The calculation of the final scores for each species was performed using the formula (1), (2), and (3) as described previously. In particular, for the second ranking list, the parameters, categories and their respective priority levels used for the calculation are shown in **Table 4**. Similar to the first selection phase, we assigned the highest priority (value = 3) to the category “Phytochemicals” and then descending values to “Growth” (value = 2) and to “Elements” (value = 1).

**Table 4:** Priority levels of categories and parameters used for the second ranking of microgreens.

Category	Category priority (P)	Parameter	Parameter priority (p)
Growth	2	Growth period	5
		Fresh yield	5
		Germination	3
		Seed volume	3
		Hypocotyl length	2
		Dry weight	2
		Sowing density	2
		L*	1
		a*	1
		b*	1
		Chroma	1
		Hue	1
Phytonutrients	3	Total Ascorbic Acid	5
		Anthocyanins	4
		Total polyphenols	4
		Lutein	3
		Total non-structural carbohydrates	2
		Total soluble carbohydrates	2
		Total chlorophylls	1
		β-carotene	1
		Violaxanthin	1
		Neoxanthin	1
		Starch	1
		Sucrose	1
		Glucose	1
		Fructose	1
Elements	1	Nitrate	1
		Sulfate	1

Overall, data on “Phytochemicals” from the cultivation tests were expressed as the quantity of the specific compound on fresh weight per day per m^2^ of cultivated area. The second phase of the selection process followed a similar approach as the first phase, in which priority levels were assigned to the parameter. The rationale of the prioritization of parameters included in the “phytochemicals” category, was based on the nutraceutical value of the specific compound, the potential loss of components in the astronaut’s physiological level during the flight, and the perishability of the compounds. Furthermore, in this second phase, statistically significant differences in the production of each compound were considered as the driving criterion for the attribution of the priority levels. Highest priority values (p = 5) were assigned to total ascorbic acid and then descending (p = 4) to anthocyanins and total polyphenols. We then assigned p = 3 to lutein and p = 2 to total non-structural carbohydrates and total soluble carbohydrates. The lowest level (p = 1) was assigned to total chlorophylls, β-carotene, violaxanthin, neoxanthin, starch, sucrose, glucose, and fructose. As regards the prioritization of “Growth” parameters, we assigned the highest priority levels to Yield, growth period (inverted), germination, and seed volume (inverted). As for parameters of the “Element” category, we assigned p = 1 for both nitrate and sulfate.

## 3 Results

### 3.1 First ranking list

The ranking list resulting from the first algorithm elaboration using literature data showed score values ranging between 0.956 and 4.687 across microgreens genotypes as shown in **Table 3**. Results showed that most genotypes belonging to the family Brassicaceae are listed at the top of the ranking. However, coriander (Apiaceae) resulted in first place with the highest score. From this ranking list we selected the six top species, namely coriander, savoy cabbage, daikon radish, red cabbage, white cabbage, and common radish, for the germination and cultivation tests of the second selection phase.

### 3.2 Germination and seed volume

**Figure 2:**
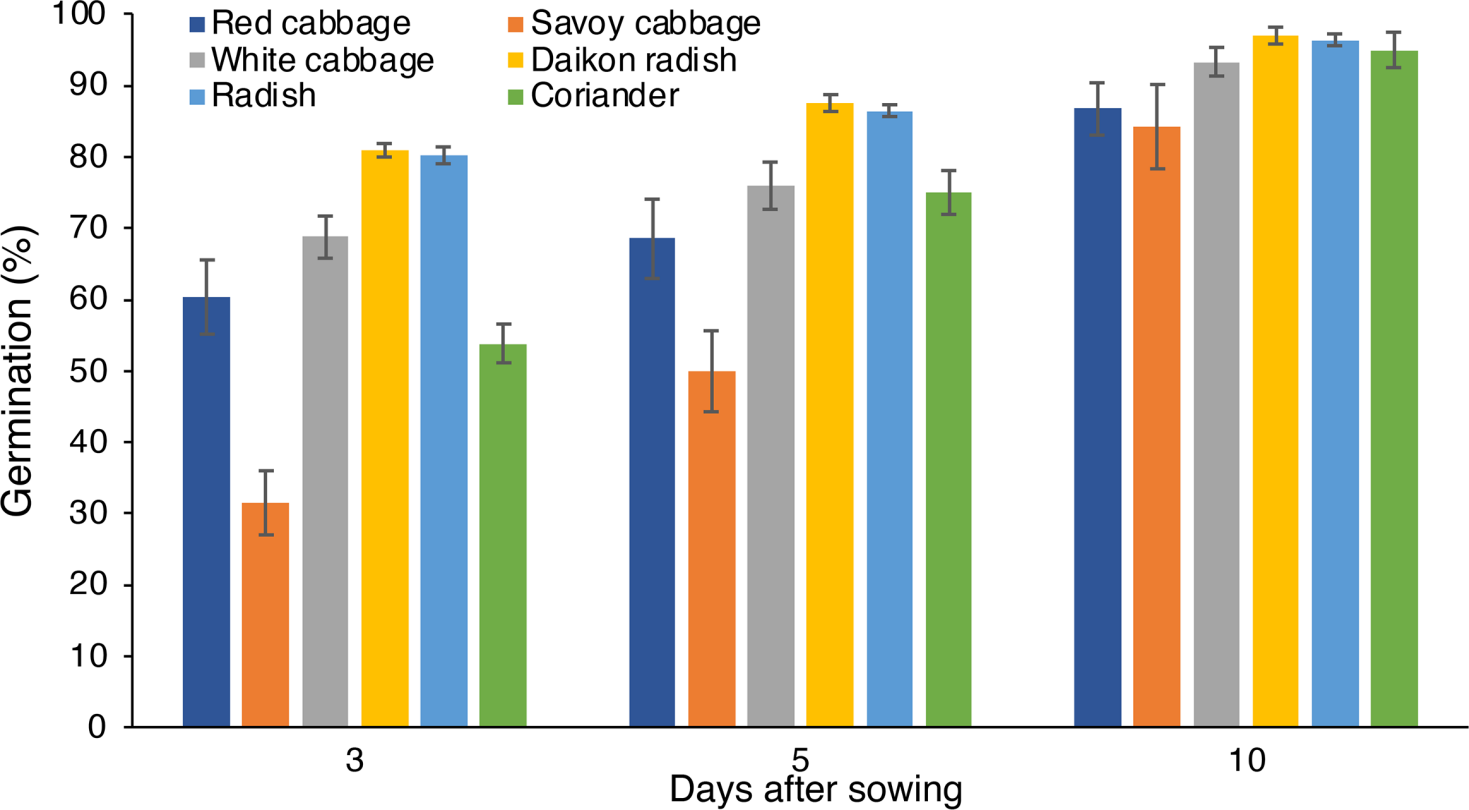
Germination of red cabbage, savoy cabbage, white cabbage, daikon radish, common radish, and coriander seeds at 3, 5 and 10 days after sowing.

Germination was higher than 50% at 3 DAS in all the species analyzed, except for savoy cabbage showing lower values (**Figure 2**). At 3 DAS, common radish and daikon radish showed the highest percentage values of 80% and 81%, respectively. Successively, the germination percentages showed an increase over time in all species up to 10 DAS, reaching maximum values between 84% (savoy cabbage) and 96% (daikon radish).

Seed volume was highest in coriander (100-Seeds, 1.4 mL), and lowest in red, savoy and white cabbage (100-Seeds, ≤ 0.55 mL), whereas 100-Seeds volume of both daikon and common radish resulted 0.65 mL (**Table S1**).

### 3.3 Results - harvest and colorimetric analysis

Among the six cultivars of microgreens used for cultivation tests, radish was characterized by the highest fresh yield and dry matter per m^2^ (3.66 kg m^-2^ and 0.25 kg m^-2^, respectively) (**Table S1**). Whereas coriander yielded the lowest (∼ 42.3% less than radish) and had the lowest hypocotyl length among all the tested microgreens, but it showed the highest DM (8.41%), that was approximately 27.15% higher than the other species. Coriander and daikon radish exhibited significantly brighter canopy (L) than red cabbage and savoy cabbage (**Table S2**). In addition, coriander canopy was greener (a*: -16.27) than the others, as opposed to red cabbage canopy that was leaning more towards red (a*: -8.65) and blue (b*: 14.61) and less saturated (Chroma: 16.98) than the other microgreens species. As for Hue, white cabbage and savoy cabbage canopy had significantly higher values than radish.

### 3.4 Results - nutritional analysis

#### 3.4.1 Non-structural carbohydrates

The production of the NSC is shown in (**Table S3**). The NSC production levels varied significantly (*p* < 0.001) among the six microgreens species. In terms of Total NSC and total soluble carbohydrates, the cultivar Candela showed a lower score compared to the other species, among which the total amount of these compounds did not vary significantly. The production levels of the soluble fractions (Glucose, Fructose, and Sucrose) resulted significantly different among the different species. Radish, savoy cabbage, white cabbage and red cabbage showed an average increase of 65% of Glucose compared to coriander and daikon radish. The highest fructose production was found in red cabbage and savoy cabbage, whereas daikon radish showed the lowest levels. For the Sucrose production, coriander showed the best performance, followed by radish and savoy cabbage. On the other hand, white cabbage, red cabbage, and daikon radish did not show significant differences, with an average value of 79.5 mg m^-2^ day^-1^ FW representing the lowest value of sucrose production. As regards the quantification of starch, the species with the highest production level was radish that showed an increase of 37%, 52%, 80%, 308% and 429% compared to red cabbage, white cabbage, savoy cabbage, coriander, and daikon radish, respectively.

#### 3.4.2 Vitamin C

Microgreens genotypes showed significant different production levels (*p* < 0.001) of Tot. Asc. A. The greatest value of Tot. Asc. A. was found in radish. The lowest level of Tot. Asc. A. was found in the cultivar coriander which showed 81% lower value compared to the best performing cultivar. Savoy cabbage, red cabbage, white cabbage and daikon radish showed intermediate values with a reduction of 18, 34, 38 and 58%, respectively, when compared to radish.

#### 3.4.3 Total anthocyanin content and total phenolic content

The content of total anthocyanins and TPC is shown in **Table 3**. The ANOVA analysis showed significant differences (*p* < 0.001) of both parameters among microgreens genotypes analyzed. The total anthocyanins content was not measurable in white cabbage and coriander. Red cabbage showed elevated accumulation of anthocyanins, resulting to be 3, 5 and 10-fold higher compared to radish, savoy cabbage and daikon radish, respectively. The best performing microgreens in terms of TPCs production resulted to be radish with 458 mg m^-2^ Day^-1^. Results also showed that red cabbage and savoy cabbage accumulated intermediate levels of TPC showing a 25%, 68% and 77% increase compared to white cabbage, daikon radish and coriander, respectively (**Table 3**).

#### 3.4.4 Pigments

The content of total chlorophylls resulted to be significantly different only for radish which showed a 57% higher production compared to the other microgreens genotypes (**Table S4**). None of the lutein, β-Carotene, neoxanthin and violaxanthin content resulted to be statistically different among the tested species, showing an overall average production of 8.7, 5.9, 2.1 and 37.3 mg m^-2^ day^-1^ FW, respectively (**Table S5**).

#### 3.4.5 Nitrate, phosphate, and sulfate

Nitrate accumulation was not detectable among the cultivars tested, whereas phosphate and sulfate accumulation resulted to be significantly different among microgreens genotypes (P<0.001) (**Table S1**). The highest level of phosphate was found in radish (367 mg m^-2^ day^-1^ FW) which also showed the highest production of sulfates (352 mg m^-2^ day^-1^ FW). On the other hand, the lowest accumulation of phosphate was found in white cabbage and coriander with an average of 182 and 179 mg m^-2^ day^1^ FW respectively, whereas red cabbage, savoy cabbage and daikon radish showed intermediate values. For the sulfate content, coriander showed the lowest accumulation with an 86% decrease compared to radish.

### 3.5 Results - calculation 2^nd^ algorithm

Experimental data gathered from the germination and cultivation tests were used to construct the working matrix for the calculation of the second ranking list including the six top species (i.e., coriander, savoy cabbage, daikon radish, red cabbage, white cabbage, and common radish). Data elaboration was performed using the priority levels of categories and parameters reported in **Table 4**. Results of the algorithm calculation showed that radish obtained the highest score (8.409), followed by savoy cabbage (6.661), whereas coriander showed the lowest score (3.957) (**Table 5**).

**Table 5:** Ranking list of the 6 top species of microgreens.

Rank	Species	Common name	Score
1	*Raphanus sativus*	Radish	8.409
2	*Brassica oleracea* var. *capitata* f. *sabauda*	Savoy cabbage	6.661
3	*Brassica oleracea* var. *capitata* f. *rubra*	Red cabbage	6.421
4	*Brassica oleracea* var. *capitata* f. *alba*	White cabbage	4.527
5	*Raphanus sativus* var. *longipinnatus*	Daikon radish	4.315
6	*Coriandrum sativum*	Coriander	3.957

## 4 Discussion

Microgreens generally have a higher content of phytonutrients and lower nitrates compared to their mature-leaf counterparts, thus resulting in high quality functional food (Xiao et al., 2012; Pinto et al., 2015). Indeed, microgreens provide a significant quantity of phytonutrients that can help astronauts in coping with the stressful conditions of the Space environment (Kyriacou et al., 2016; Kyriacou et al., 2017).

Since there are many species that have been proposed for microgreens production (Kyriacou et al., 2016), we developed a method based on an algorithm with the aim of objectively select those microgreens species most suitable for cultivation in Space according to productivity and phytonutrient profile criteria. The selection method consisted of two subsequent rankings elaborated using first literature data and, successively, using data obtained with cultivation tests specifically conducted for this study. The first ranking list included 39 plant species commonly used for the production of microgreens according to the reference literature. Most of the species herein considered belong to the family Brassicaceae (26 out of 39) whereas the remaining species belong to Amaranthaceae (1), Apiaceae (2), Chenopodiaceae (3), Fabaceae (1), Lamiaceae (2), Malvaceae (1), Poaceae (1), Polygonaceae (2). In this regard, although there is a growing interest in microgreens and in the use of new microgreens varieties, no studies presented a comparison between species for the selection of microgreens genotypes based on productivity and phytonutrient profile criteria. Indeed, most studies on microgreens investigated the effects of environmental conditions or cultivation techniques using one or a few species. For this study, in the first selection phase, we focused on documents providing a comparison between different microgreens species grown under standard conditions. With this approach we gathered literature data on horticultural and nutritional parameters to be included in the working matrix for a comparative analysis between the different species based on the prioritization of the different parameters.

A similar approach based on parameter prioritization has been used by De Micco et al. (2012) and Massa et al. (2015), but with differences from our study mostly due to the specific aims of the selection. Indeed, prioritization of parameters can have a large effect on the final scores of the different species. Nevertheless, the method herein reported provides the possibility of modeling species selection by adjusting the prioritization of productivity and phytonutrient parameters according to specific objectives. In our case, the selection process aimed to ensure that the selected plant food had elevated nutraceutical value.

After the first selection phase we identified six top species with the highest score that were then used for germination and cultivation tests. This restricted list of microgreens allowed us to study each species with more details introducing new parameters to be measured and implemented in the working matrix for the elaboration of the ranking list. For this second selection phase, we chose to include in the working matrix data of “Phytonutrients” parameters expressed as the daily production per m^2^. This approach allowed us to better identify those species with higher production efficiency of phytonutrients, considering there are constraints regarding volume available for cultivation in Space. Furthermore, data on daily production of phytonutrients per m^2^ are useful to estimate the surface to be cultivated according to the growth period of each species and the astronauts’ daily dietary requirements.

Ghoora et al. (2020) investigated the contribution of nutrients (NC), compared to the nutrient claims of FDA (U.S. Food and Drug Administration), of ten culinary microgreens with a consumption of 85g (USDA reference amount customarily consumed of green leafy vegetables) of fresh biomass. Authors pointed out that the vitamin’s NC ranged between 28.2–116.4%, 28.5–332% and 24.3–71.8% respectively for ascorbic acid (vitamin C), α-tocopherol (vitamin E) and β-carotene (pro-vitamin A). Since microgreens can efficiently fulfill the daily intakes of vitamins, along with a great contribution in antioxidants, the prioritization of different categories favored the “Phytonutrients” category. However, growth characteristics that describe the general productivity efficiency of the crop, such as yield, dry weight, and growth period, cannot be understated since they impact the overall size of the cropping system, thereby affecting key aspects of Space facilities as total resources used and mass at launch. For these reasons, we assigned the second highest priority level to the “Growth” category. Pinto et al. (2015) investigated the potential contribution of lettuce microgreens to supply the recommended daily intake of minerals. In the study by Pinto et al. (2015), the Ca, P, Mg, K, and Na Estimated Daily Intake (EDI), based on a consumption of 25 g of microgreens, respectively represented only the 3.48, 3.10, 1.76, 1.75, 0.83% of the EFSA RDAs (recommended dietary allowances) and AIs (adequate intakes) mean for men and women. In the study by Ghoora et al. (2020) the NC of macro-elements such as Ca, Mg, K, Na reached respectively 6.2%, 14.4%, 8.7% and 8.4%. In our view, producing food rich in minerals *via* microgreens is less relevant than producing bioactive molecules, since minerals are stable during conservation. Furthermore, even if microgreens can contribute to the mineral supply, the achievement of the recommended daily intake solely through the consumption of microgreens is difficult, while it can be easily obtained using other food sources. Therefore, we assigned to “Elements” category the lowest priority level.

Maintaining proper antioxidant health is a priority for astronauts during spaceflight. Several factors including ionizing radiation, microgravity, exercise and even diet are a source of ROS (Powers and Jackson, 2008; Azzam et al., 2012; Zwart et al., 2013; Ran et al., 2016). The role of phytochemicals such as vitamins and polyphenols in maintaining antioxidant health have been reviewed by several authors (Smith et al., 2014; Heer et al., 2020; Gómez et al., 2021; Kulkarni et al., 2022). The protective effect of Vitamin A, C, and E against γ-rays was investigated *in vitro* and the potential to reduce DNA damages before and after irradiation was shown (Konopacka and Rzeszowska-Wolny, 2001). In addition, the radioprotective potential of both vitamin A and C were successfully tested in mice (Mortazavi et al., 2015; Changizi et al., 2019). These compounds also have a synergistic effect by enhancing the antioxidant potential when administered together (Tauler et al., 2002). Moreover, vitamin A, C and E are involved in several fundamental physiological processes in the human body such as immune function, bone health, eye health, and are related to the body’s response to the space environment (Smith et al., 2014).

In order to give priority to a specific nutraceutical component useful for the astronauts’ diet, it is necessary to evaluate the deficiencies that may occur during the spaceflight. Smith et al. (2005) analyzed the blood levels of vitamins and minerals in 11 astronauts before and after 128-195 day of spaceflight on board of the International Space Station. They pointed out that vitamin E was affected by the spaceflight since γ-tocopherol significantly decreased after landing. However, no losses were detected for β-carotene, whereas the ascorbate status has not yet been investigated. As regards the stability of the nutraceuticals in Space-stored food, while vitamin A declines but with sufficient concentrations kept after 3 years of storage, the vitamin C content in most fruit products degrades between 32 and 83% (Cooper et al., 2017). Moreover, Vitamin E has been found to be the most sensitive to irradiation during storage (Watkins et al., 2022). According to the aforementioned criteria, the highest priority among the parameters of the “Phytonutrient” category, in the first selection phase, was assigned to ascorbic acid, tocopherols, β-carotene and polyphenols.

Bone loss is a significant health concern associated with long-duration Space missions and the microgravity environment (Leblanc et al., 2007). The rate of bone mineral loss during spaceflight is estimated to be between 0.5-1% per month (Weaver, 2000). Net rate of bone calcium loss in Mir and Skylab missions resulted in approximately 250 mg every day. Adequate intake of calcium-rich foods or supplements will play a significant role in ensuring the skeletal health of astronauts during prolonged Space missions (Smith et al., 2005). In this regard, the highest priority level for parameters included in the “Element” category was assigned to calcium.

As regards iron and nitrate parameters included in the “Element” category, data were inverted in order to valorize microgreens genotypes with low accumulation. Low iron and nitrate accumulator species are desirable when selecting crops for BLSSs. The International Space Station (ISS) food system has high levels of dietary iron. The current iron U.S. Dietary Reference Intake (DRI) is 8-10 mg d^-1^ and crew members on long-duration Space missions have been observed to consume excessive amounts of iron (20-25 mg iron d^-1^) (Smith and Zwart, 2008). In addition, physiological changes during spaceflight led to an accumulation of iron in storage tissues which can act as an oxidizing agent inducing damages at DNA level (Glei et al., 2002). In addition, high amounts of nitrate can cause serious health problems for humans. The accumulation of nitrate in leafy crops has been largely reported (Maynard et al., 1976; Colla et al., 2018). The European Commission Regulation No 1258/2011 establishes the limits for nitrate content in vegetable productions based on species and growing conditions. Importantly, despite microgreens are young plantlets with low incidence of nitrate accumulation (Pinto et al., 2015), the cultivation practices could influence the content of this compound (Bulgari et al., 2021; Kyriacou et al., 2021).

Overall, according to parameters prioritization discussed above and the algorithm calculation, radish and savoy cabbage are the most suitable microgreens genotypes to be used for cultivation in Space. Nevertheless, with the possibility to adjust the algorithm calculation based on productivity and phytonutrient profile criteria, further research deepening the effects of microgravity on human’s metabolism, and the related countermeasures based on specific diets, can provide data and insights to optimize the selection of plant food species intended for cultivation in Space. Horticultural research, on the other hand, can provide strategies for maximizing the production of certain characteristics.

## 5 Conclusions

The selection method of microgreens developed in this study uses estimated scores based on prioritization of parameters and is preferred over a simple listing of plants since it has an argued basis for the given priorities. The method herein reported consists of two subsequent rankings elaborated using first literature data and, successively, using data obtained with cultivation tests specifically conducted for this study. The method also provides the possibility to adjust the algorithm calculation and can be repeated for further selection including new crops or new data. Furthermore, the approach of grouping the different parameters into different categories allow to easily assign higher or lower priorities related to other parameters included in the same group, instead of prioritizing parameters all together. So far, the flexibility of this selection method provides potential adaptability for selecting new cultivars and/or including other criteria relevant to support astronauts’ diet for different aims such as growth chambers on orbital platforms, long-duration travels, or extraterrestrial colonization.

## Data availability statement

The original contributions presented in the study are included in the article/**Supplementary Material**. Further inquiries can be directed to the corresponding authors.

## Author contributions

LI and GA conceived the paper. All authors contributed to general planning and data analyses, LI, CN and GP developed a first draft of the manuscript. All authors provided constructive discussions, revised, and approved the submitted version of the article.
