# Supplementary material for: Applying productivity and phytonutrient profile criteria in modelling species selection of microgreens as Space crops for astronaut consumption
Source: Front Plant Sci. 2023 Aug 11;14:1210566. doi: 10.3389/fpls.2023.1210566 (PMC10450622; doi:10.3389/fpls.2023.1210566)
Supplement: Supplementary file 1 [file Table_1.docx]

Supplementary Material

Applying productivity and phytonutrient profile criteria in modelling species selection of microgreens as Space crops for astronaut consumption

Luigi Gennaro Izzo^1^, Christophe El Nakhel^1,*^, Youssef Rouphael^1^, Simona Proietti^2^, Gabriele Paglialunga^2,*^, Stefano Moscatello^2^, Alberto Battistelli^2^, Maurizio Iovane^1^, Leone Ermes Romano^1^, Stefania De Pascale^1^, Giovanna Aronne^1^

^1^Department of Agricultural Sciences, University of Naples Federico II, Portici, Italy

^2^National Research Council of Italy, Research Institute on Terrestrial Ecosystems, Porano, Italy

*** Correspondence:** christophe.elnakhel@unina.it; gabriele.paglialunga@iret.cnr.it

Table S1. Biometric parameters, and Phosphate and Sulfate content

| Source of Variance | 100-Seeds volume | Hypocotyl length | Fresh yield | Dry biomass | DM | Phosphate | Sulfate |
| --- | --- | --- | --- | --- | --- | --- | --- |
|  | mL | cm | kg m^-2^ | | % | mg m^-2^ day^-1^ FW | |
| **Species** |  |  |  |  |  |  |  |
| Daikon radish | 0.65 | 6.23 ± 0.18 a | 2.09 ± 0.04 d | 0.13 ± 0.01 d | 6.33 ± 0.13 b | 239 ± 13 b | 271 ± 14 b |
| White cabbage | 0.50 | 5.97 ± 0.02 a | 2.43 ± 0.03 c | 0.16 ± 0.01 cd | 6.44 ± 0.40 b | 182 ± 27 b | 288 ± 23 ab |
| Coriander | 1.40 | 4.61 ± 0.12 b | 2.11 ± 0.02 d | 0.18 ± 0.00 bc | 8.41 ± 0.09 a | 179 ± 18 b | 50.3 ± 3.0 c |
| Red cabbage | 0.55 | 5.84 ± 0.30 a | 2.71 ± 0.04 b | 0.18 ± 0.00 b | 6.74 ± 0.08 b | 218 ± 1 b | 293 ± 20 ab |
| Radish | 0.65 | 6.36 ± 0.23 a | 3.66 ± 0.09 a | 0.25 ± 0.01 a | 6.89 ± 0.15 b | 367 ± 14 a | 352 ± 5.9 a |
| Savoy cabbage | 0.45 | 6.63 ± 0.08 a | 2.84 ± 0.03 b | 0.19 ± 0.00 b | 6.67 ± 0.04 b | 196 ± 15 b | 333 ± 15 ab |
| Significance |  | *** | *** | *** | *** | *** | *** |

*** Significant at *p* < 0.001. Different letters within each column indicate significant differences according to Tukey’s multiple-range test (*p* = 0.05)

**Table S2. Colorimetric parameters**

| Source of Variance | L* | a* | b* | Chroma | Hue |
| --- | --- | --- | --- | --- | --- |
|  |  |  |  |  |  |
| **Species** |  |  |  |  |  |
| Daikon radish | 41.28 ± 2.29 a | -14.27 ± 0.56 d | 23.71 ± 0.71 b | 27.68 ± 0.88 b | 121.0 ± 0.4 ab |
| White cabbage | 37.40 ± 0.95 ab | -12.42 ± 0.14 b | 20.26 ± 0.60 c | 23.77 ± 0.57 c | 121.6 ± 0.5 a |
| Coriander | 42.11 ± 0.99 a | -16.27 ± 0.08 e | 27.34 ± 0.34 a | 31.82 ± 0.33 a | 120.8 ± 0.2 ab |
| Red cabbage | 33.15 ± 0.69 b | -8.65 ± 0.11 a | 14.61 ± 0.14 d | 16.98 ± 0.13 d | 120.6 ± 0.4 ab |
| Radish | 38.27 ± 0.16 ab | -13.7 ± 0.24 cd | 24.02 ± 0.31 b | 27.66 ± 0.35 b | 119.7 ± 0.4 b |
| Savoy cabbage | 34.68 ± 0.51 b | -12.51 ± 0.08 bc | 19.98 ± 0.05 c | 23.58 ± 0.05 c | 122.1 ± 0.2 a |
| Significance | *** | *** | *** | *** | * |

*, *** Significant at *p* < 0.05 and 0.001, respectively. Different letters within each column indicate significant differences according to Duncan Tukey’s multiple-range test (*p* = 0.05)

**Table S3. Sugars and starch accumulation, total soluble carbohydrates (TSC), and total non-structural carbohydrates (NSC)**

| Source of Variance | Glucose | Fructose | Sucrose | Starch | TSC | NSC |
| --- | --- | --- | --- | --- | --- | --- |
|  | mg m^-2^ day^-1^ FW | | | | | |
| **Species** |  |  |  |  |  |  |
| Daikon radish | 706 ± 91 c | 160 ± 22 d | 77.8 ± 14 d | 105 ± 25 d | 943 ± 125 b | 1048 ± 145 b |
| White cabbage | 1175 ± 17 ab | 234 ± 18 cd | 80.7 ± 1 cd | 364 ± 56 abc | 1484 ± 48 ab | 1982 ± 192 a |
| Coriander | 814 ± 33 bc | 395 ± 12 ab | 864 ± 25 a | 136 ± 8 cd | 2073 ± 51 a | 2209 ± 46 a |
| Red cabbage | 1170 ± 52 ab | 460 ± 43 ab | 80.2 ± 5 cd | 406 ± 91 ab | 1710 ± 93 a | 2116 ± 183 a |
| Radish | 1433 ± 143 a | 325 ± 41 bc | 236 ± 2 b | 555 ± 17 a | 1994 ± 182 a | 2549 ± 193 a |
| Savoy cabbage | 1218 ± 80 ab | 491 ± 41 a | 136 ± 3 c | 308 ± 55 bcd | 1846 ± 122 a | 2154 ± 174 a |
| Significance | *** | *** | *** | *** | *** | *** |

*** Significant at *p* < 0.001. Different letters within each column indicate significant differences according to Tukey’s multiple-range test (*p* = 0.05). TSC = glucose + fructose + sucrose; NSC = TSC + starch

Table S4. Pigments, Total Phenolic Content (TPC) and Total Ascorbic Acid (Tot. Asc.A.) content

| Source of Variance | Total chlorophyll | Anthocyanins | TPC | Tot. Asc. A |
| --- | --- | --- | --- | --- |
|  | mg m^-2^ day^-1^ FW | | | |
| **Species** |  |  |  |  |
| Daikon radish | 30.64 ± 2.91 b | 1.55 ± 0.11 c | 200 ± 9 d | 79.5 ± 4 cd |
| White cabbage | 26.36 ± 2.34 b | N.D. | 268 ± 22 c | 104 ± 8 bc |
| Coriander | 33.74 ± 2.55 b | N.D. | 189 ± 6 d | 31.9 ± 2 d |
| Red cabbage | 37.05 ± 0.16 ab | 14.9 ± 1.13 a | 347 ± 17 b | 111 ± 15 bc |
| Radish | 49.77 ± 5.50 a | 4.98 ± 0.85 b | 458 ± 12 a | 168 ± 21 a |
| Savoy cabbage | 31.39 ± 3.79 b | 2.74 ± 0.05 bc | 323 ± 14 bc | 138 ± 11 ab |
| Significance | ** | *** | *** | *** |

**, *** Significant at *p* < 0.01 and 0.001, respectively. Different letters within each column indicate significant differences according to Tukey’s multiple-range test (*p* = 0.05). N.D. = not detected.

Table S5. Lutein, β-carotene, Neoxanthin and Violaxanthin content

| Source of Variance | Lutein | β-Carotene | Neoxanthin | Violaxanthin |
| --- | --- | --- | --- | --- |
|  | mg m^-2^ day^-1^ FW | | | |
| **Species** |  |  |  |  |
| Daikon radish | 8.51 ± 1.46 | 6.29 ± 0.85 | 2.24 ± 0.23 | 50.3 ± 10 |
| White cabbage | 6.29 ± 1.59 | 4.26 ± 0.41 | 1.44 ± 0.01 | 22.3 ± 5.5 |
| Coriander | 6.74 ± 0.47 | 4.92 ± 0.47 | 2.10 ± 0.23 | 34.8 ± 3.1 |
| Red cabbage | 8.58 ± 1.26 | 6.27 ± 1.32 | 1.67 ± 0.28 | 34.7 ± 8.1 |
| Radish | 10.3 ± 0.88 | 6.13 ± 0.26 | 2.37 ± 0.53 | 49.7 ± 5.7 |
| Savoy cabbage | 11.5 ± 3.16 | 7.38 ± 2.16 | 2.55 ± 0.66 | 31.7 ± 4.9 |
| Significance | ns | ns | ns | ns |

Ns = Non significant. Different letters within each column indicate significant differences according to Tukey’s multiple-range test (*p* = 0.05)
